# Bioisosteric Matrices for Ligands of Serotonin Receptors

**DOI:** 10.1002/cmdc.201402563

**Published:** 2015-03-13

**Authors:** Dawid Warszycki, Stefan Mordalski, Jakub Staroń, Andrzej J Bojarski

**Affiliations:** [a] Institute of Pharmacology, Polish Academy of Sciences12 Smetna Street, 31-343 Kraków (Poland)

**Keywords:** bioisosterism, bioisosteric matrices, bioisosteric substitutions, chemical space, serotonin receptors

## Abstract

The concept of bioisosteric replacement matrices is applied to explore the chemical space of serotonin receptor ligands, aiming to determine the most efficient ways of manipulating the affinity for all 5-HT receptor subtypes. Analysis of a collection of over 1 million bioisosteres of compounds with measured activity towards serotonin receptors revealed that an average of 31 % of the ligands for each target are mutual bioisosteres. In addition, the collected dataset allowed the development of bioisosteric matrices—qualitative and quantitative descriptions of the biological effects of each predefined type of bioisosteric substitution, providing favored paths of modifying the compounds. The concept exemplified here for serotonin receptor ligands can likely be more broadly applied to other target classes, thus representing a useful guide for medicinal chemists designing novel ligands.

Bioisosteric replacement is a technique of transforming a compound by exchanging a group of atoms for a different group that is similar in terms of its physicochemical properties. The underlying purpose of this modification is the enhancement of a certain biological or chemical property, such as affinity towards a given target, pharmacodynamics, pharmacokinetics, or the exploration of new, unknown scaffolds. Although fundamentally simple, the method has been successfully applied in numerous drug design projects and has led to some spectacular results, such as the discovery of prontosil (its active metabolite, sulfanilamide, is a bioisostere of *para*-aminobenzoic acid)[1] and pindolol (a nonselective beta blocker, developed from propranolol by replacing the naphthalene moiety with indole). Given its capabilities, the full potential of bioisosterism has not yet been utilized, especially in the field of serotonin receptor ligands; to the best of our knowledge, only four reports in the literature explicitly applying the strategy of bioisosteric replacement.[2–5]

In May 2013, approximately 22 000 unique ligands with known activity against any serotonin receptor were annotated in the ChEMBL database.[6] Despite this quantity and the relatively high diversity within this set, the vast majority of compounds contain two typical pharmacophore features characteristic of serotonergic ligands: an aromatic system and a polarizable nitrogen atom. Following the classification of 5-HT receptor ligands described in the literature, ten key structural classes of ligands can be highlighted: tryptamines, phenylalkylamines, aminotetralines, aporphines, ergolines, arylpiperazines, piperidines, tricycles, indole and sulfonamide derivatives;[7–16] these classes were used in this research.

To apply the concept of bioisosterism to serotonin receptors, we conducted a detailed exploration of the bioisosteric data available. The methodology of using substitution matrices, as demonstrated here for 5-HT_6_R, allows the identification of the most frequent and most efficient replacements in terms of biological activity and provides valuable suggestions for modifications to existing ligands for the rational design of new, potent and selective ligands for serotonin receptors.

The source of the compounds was the ChEMBL database version 16 (May 2013). To preserve the coherence of the data, only compounds with defined *K*_i_ values or equivalent (IC_50_, assumed to be 2×*K*_i_; p*K*_i_ and pIC_50_ values were converted to *K*_i_), as assayed on human cloned, rat cloned or native receptors, were considered. In the case of multiple data for a single ligand, the *K*_i_ value and data against human receptors were preferred; a median value was used in the case of multiple biological results. Among the 14 members of the 5-HTR family, two were rejected: 5-HT_3_R because it is an ion channel and 5-HT_5B_R due to an insufficient number of ligands to perform a comprehensive analysis. The set of 5-HT_6_R ligands fetched from the ChEMBL database (2277 compounds) was enriched by data extracted from approximately 40 patents (2529 compounds). For each compound acting on the remaining 5-HTR subtypes (5-HT_1A_, 5-HT_1B_, 5-HT_1D_, 5-HT_1E_, 5-HT_1F_, 5-HT_2A_, 5-HT_2B_, 5-HT_2C_, 5-HT_4_, 5-HT_5A_, 5-HT_6_, 5-HT_7_), all classic bioisosteric replacements implemented in Pipeline Pilot[17] (divided into six classes: ring, amide, carbonyl, halogen and hydroxyl substitutions and ring modifications) were applied. The input structures and duplicates were removed from the original collections, leading to 12 sets of unique bioisosteres. Each compound was then queried against all other sets (regardless of target). Finding the same structure in the ligand and bioisostere set indicated that bioisostere replacement between those repositories exists. This procedure was repeated for all bioisostere collections and led to the identification of all bioisosteres between all targets as well as within the chemical space of single receptor (self-bioisosteres) (Table 1). The collections of bioisosteres belonging to the same class of ligands were gathered into matrices, revealing the amount of positive, neutral and negative substitutions in terms of affinity towards a given target.

**Table 1:** The number of bioisosteres found for different serotonin receptor subtypes.

Receptor	5-HT_1A_	5-HT_1B_	5-HT_1D_	5-HT_1E_	5-HT_1F_	5-HT_2A_	5-HT_2B_	5-HT_2C_	5-HT_4_	5-HT_5A_	5-HT_6_	5-HT_7_
5-HT_1A_	2053	204	248	27	48	384	60	156	30	34	113	165
5-HT_1B_	204	316	297	29	51	60	40	64	23	22	75	59
5-HT_1D_	248	297	378	32	52	72	47	75	31	25	89	66
5-HT_1E_	27	29	32	26	13	27	18	27	22	19	35	28
5-HT_1F_	48	51	52	13	61	13	13	13	9	3	27	15
5-HT_2A_	384	60	72	27	13	1306	288	818	25	61	129	103
5-HT_2B_	60	40	47	18	13	288	341	282	19	13	59	42
5-HT_2C_	156	64	75	27	13	818	282	921	25	30	140	105
5-HT_4_	30	23	31	22	9	25	19	25	130	15	28	19
5-HT_5A_	34	22	25	19	3	31	13	30	15	39	31	23
5-HT_6_	113	75	89	35	27	129	59	140	28	31	2180	131
5-HT_7_	165	59	66	28	15	103	42	105	19	23	131	347

Self-bioisosteres represent the largest portion of the analyzed population of ligands (with the exception of 5-HT_1E_R). Unsurprisingly, the number of self-bioisosteres is proportional to the quantity of ligands (Table 2). The highest rate of self-bioisosteres was observed for the 5-HT_1F_R and 5-HT_6_R, due to enrichment of the original sets retrieved from ChEMBL with patent data (5-HT_6_R) and to exploration of structure—activity relationship studies in a limited number of published papers (5-HT_1F_R). A relatively high number of bioisosteres is also observed for closely related targets (e.g., 5-HT_2_Rs).

**Table 2:** Abundance of types of bioisosteric replacements by class.

Receptor	Ligands	Bioisosteres	Self-bioisosteres[a]			Replacement class				Total
				Ring biois[b]	Amide	Carbonyl	Halogen	Hydroxyl	Ring mod.[c]	
5-HT_1A_	6709	293477	0.306	362	142	108	692	14	735	2053
5-HT_1B_	1145	50084	0.276	66	44	48	82	2	74	316
5-HT_1D_	1319	59318	0.287	68	64	46	90	2	108	378
5-HT_1E_	132	5905	0.197	0	0	0	16	0	10	26
5-HT_1F_	125	5868	0.488	20	14	0	6	2	19	61
5-HT_2A_	3864	167910	0.338	114	18	16	804	2	352	1306
5-HT_2B_	1017	44248	0.335	34	8	10	214	2	73	341
5-HT_2C_	3019	126863	0.305	56	30	26	596	2	211	921
5-HT_4_	532	36453	0.244	8	24	2	58	0	38	130
5-HT_5A_	271	8453	0.144	2	2	2	26	0	7	39
5-HT_6_	4806	165675	0.454	278	26	142	1054	2	678	2180
5-HT_7_	3019	64212	0.242	52	12	24	154	4	101	347

[a] Fraction of self-bioisosteres;

[b] Substitution of the entire ring systems;

[c] Ring modifications only (e.g., opening, contraction, etc.).

The most frequent replacements for self-bioisosteres are the following: halogen replacements (nearly 50 % of all substitutions), ring modifications, and substitutions. Apparently, the hydroxyl class of substitutions was the least frequently explored within the investigated dataset. The analysis of bioisosteric substitutions for 5-HT_6_R ligands showed two types of substitutions that enhanced activity: 2-substituted pyridine ring substituted by other aromatic systems, especially phenyl (Table 3), and nitrile group, which are interchangeable with any halogen (Figure 1). Moreover, the introduction of sulfonamide (Table 4) and urea increases affinity towards 5-HT_6_R in all cases.

**Table 3:** Affinity of compounds for the 5-HT_6_R, depending on the presence of a 2-pyridine or a phenyl group.

Compd	R=2-pyridine	R=Ph				Compd	R=2-pyridine					R=Ph
	*K*_i_ [nm]	IC_50_ [nm]	*K*_i_ [nm]	IC_50_ [nm]			*K*_i_ [nm]	IC_50_ [nm]	*K*_i_ [nm]	IC_50_ [nm]
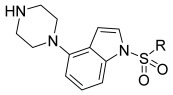	1[19]	–	1[20]	*–*		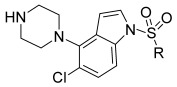	–	860[22]	2[23]	*–*
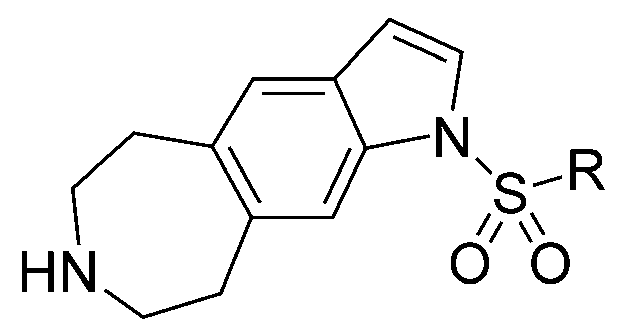	0.5[19]	–	1[19]	*–*		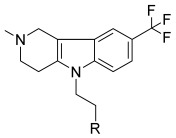	–	15750[22]	100[23]	*–*
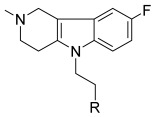	100[21]	–	1.58[21]	*–*		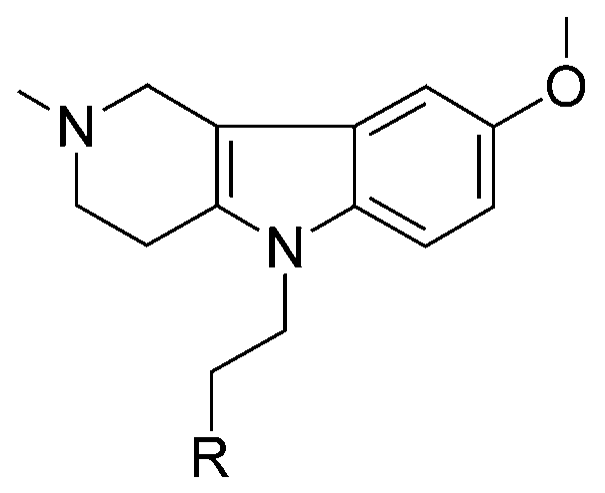	10[24]	–	9.6[24]	*–*
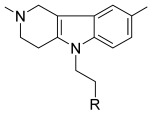	–	1590[22]	–	974[23]		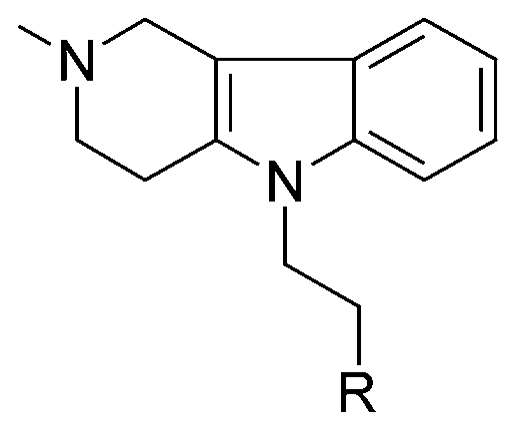	1054[25]	–	0.5[26]	*–*
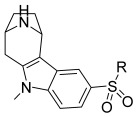	–	2980[22]	–	692[23]		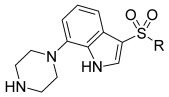	–	25300[27]	–	4940[27]
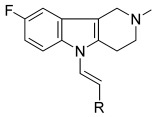	–	8360[22]	–	1057[23]		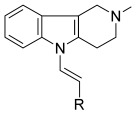	–	45900[27]	–	3790[27]

**Figure 1:**
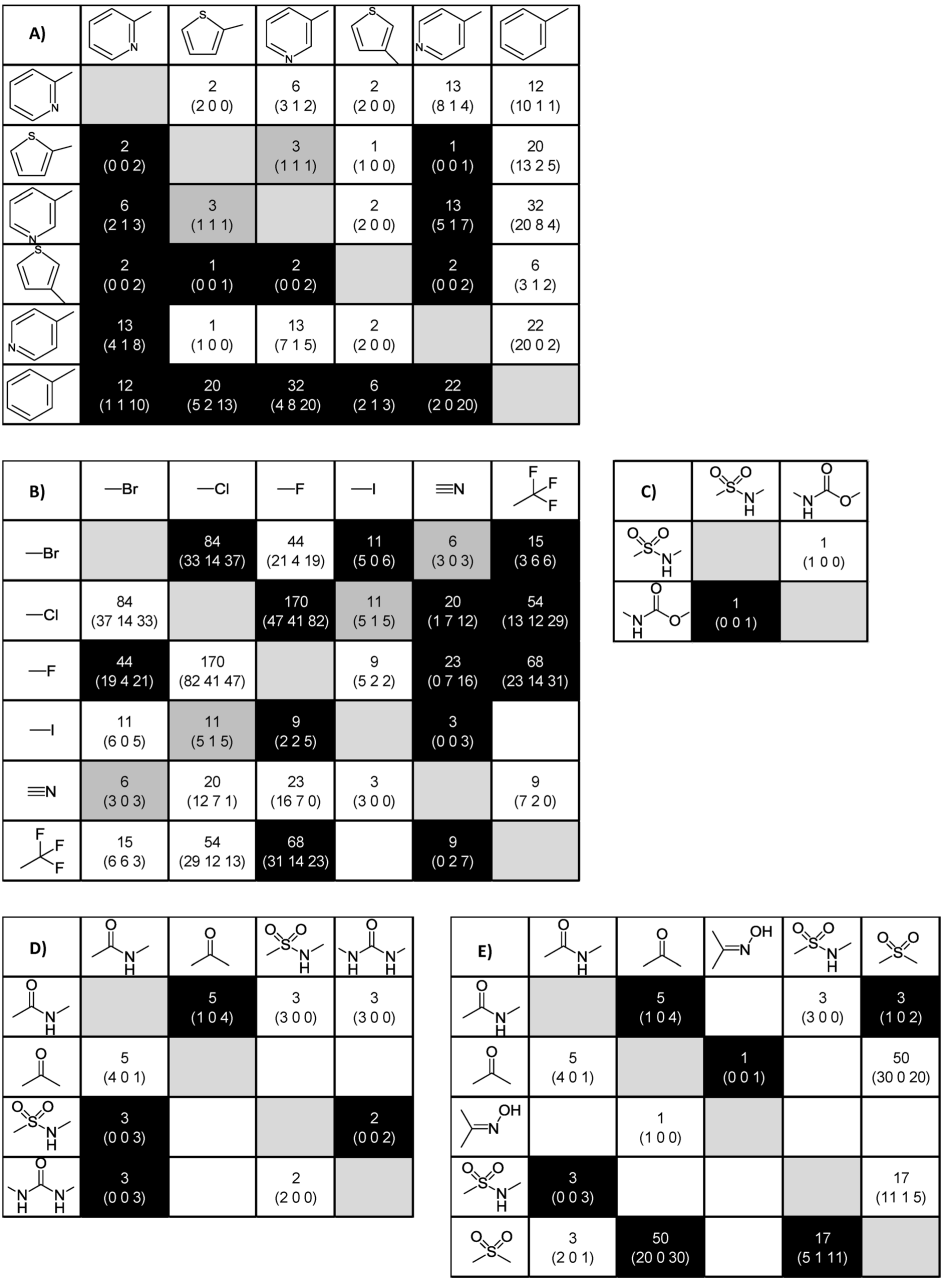
All bioisosteric replacements for 5-HT_6_R ligands belonging to A) halogen, B) phenyl, C) hydroxyl, D) amide, and E) carbonyl modifications. The total number of a given replacement is given in the intersection field. The three numbers in parentheses represent the number of replacements that increase (X _ _), do not change (_ X _) and decrease (_ _ X) the affinity. Color code: desirable substitutions (□); substitutions that decrease activity (▪); substitutions that do not influence activity (▪).

**Table 4:** Affinity of compounds for the 5-HT_6_R, depending on the presence of an amide or a sulfonamide functionality.

Compd	*K*_i_ [nm]	
	X=CO	X=SO_2_
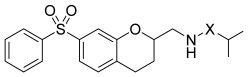	3981[28]	2512[28]
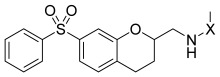	4.4[29]	0.4[29]
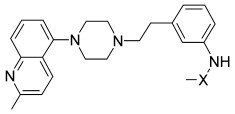	3.0[29]	0.3[29]

Analogous analysis for 5-HT_1A_R ligands proves that the introduction of 2-pyridine or replacement of the trifluoromethyl group usually leads to increased affinity (see Supporting Information). For 5-HT_2A_R ligands, 2-pyridine, 2-thiophene, nitrile and trifluoromethyl are undesirable, and the removal of a bromo substituent negatively affects the activity. On the contrary, substitutions introducing a bromo group are the most desirable for the modulation of activity towards 5-HT_2C_R. 2-Pyridine, nitrile and iodo should be replaced similarly to other targets. An amide moiety is preferred over a nitrile in potent 5-HT_4_R ligands. For 5-HT_7_R ligands, substitution of phenyl or 2-thiophene increases activity, as does replacement of the amide linker, especially with sulfonamide.[18]

Here, some restrictions were applied to investigate the replacement of groups, with the knowledge that different targets might have different activity thresholds. For each receptor–receptor pair, only substitutions appearing at least five times in the global analysis and with at least twofold more examples of increasing affinity than decreasing (or vice versa) were considered. In general, ring modifications (linearization or expansion) decreased activity towards all targets. Substitution of an amide with a carbonyl moiety in 5-HT_1A_R ligands enhances affinity towards 5-HT_1B_R and 5-HT_1D_R but decreases affinity toward 5-HT_7_R. Transition from amide to sulfonamide or urea shifts activity towards 5-HT_1B_R and 5-HT_1D_R, respectively, in the majority of cases. Conversely, reverse substitution (sulfonamide to amide) in 5-HT_1A_R ligands enhances the activity of compounds against subtypes 1D and 2A. Exchanging a fluoro with a chloro group in 5-HT_6_R ligands increases the activity for receptors 5-HT_1A_, 5-HT_1B_ and 5-HT_1D_, whereas a chloro group in 5-HT_1A_R ligands replaced with other halogens leads to decreased affinity towards 1B, 1D and 2C subtypes. All ring modifications of 5-HT_1A_R ligands decrease the affinity for 5-HT_1B_R, 5-HT_1D_R and 5-HT_1F_R and 5-HT_2_R.

We observed that the modification of 5-HT_1B_R ligands results in highly potent 5-HT_1D_R compounds, as shown with 16 different substitutions. Moreover, ring modifications in these ligands increase activity towards 5-HT_1F_R, 5-HT_2_Rs and 5-HT_7_R subtypes. Ring contraction of 5-HT_1D_, 5-HT_1E_ and 5-HT_1F_ receptors ligands leads to more active 5-HT_7_R ligands as well; however, ring expansion in 5-HT_1D_R ligands decreases activity towards 5-HT_2_Rs. A bromo group in place of other halogens in 5-HT_2A_R ligands enhances the affinity towards 5-HT_2B_R. An identical effect is achieved if a fluoro group is substituted with another halogen; this replacement generates compounds that are active toward 5-HT_2C_R and 5-HT_7_R.

In summary, the concept of bioisosteric matrices has been applied to analyze the chemical space of ligands of the serotonin receptors. The results revealed that in many cases, the given ligand is a bioisostere of a compound active towards a different member of the 5-HTR family. The number of such cases allows the assumption that bioisosteric replacement could be a viable method for discovering novel, promising ligands for serotonin receptors. This statement is also supported by a large number (30 % of the population on average) of self-bioisosteres. The size of this set results from extensive structure–activity relationship studies, exploring a significant number of substitutions. However, the trend to replace terminal and relatively simple groups indicates that the full potential of the bioisosteric substitution has yet to be revealed. All bioisosteres analyzed in this study are available from the authors upon request.
