# Supplementary material for: Bioisosteric Matrices for Ligands of Serotonin Receptors
Source: ChemMedChem. 2015 Mar 13;10(4):601–5. doi: 10.1002/cmdc.201402563 (PMC4471634; doi:10.1002/cmdc.201402563)
Supplement: Supplementary file 1 [file cmdc0010-0601-sd1.pdf]

CHEMMEDCHEM
Chemistry & Drug Discovery

Supporting Information

Bioisosteric Matrices for Ligands of Serotonin Receptors

Dawid Warszycki, Stefan Mordalski, Jakub Staroń, and Andrzej J. Bojarski*[a]

cmdc_201402563_sm_miscellaneous_information.pdf

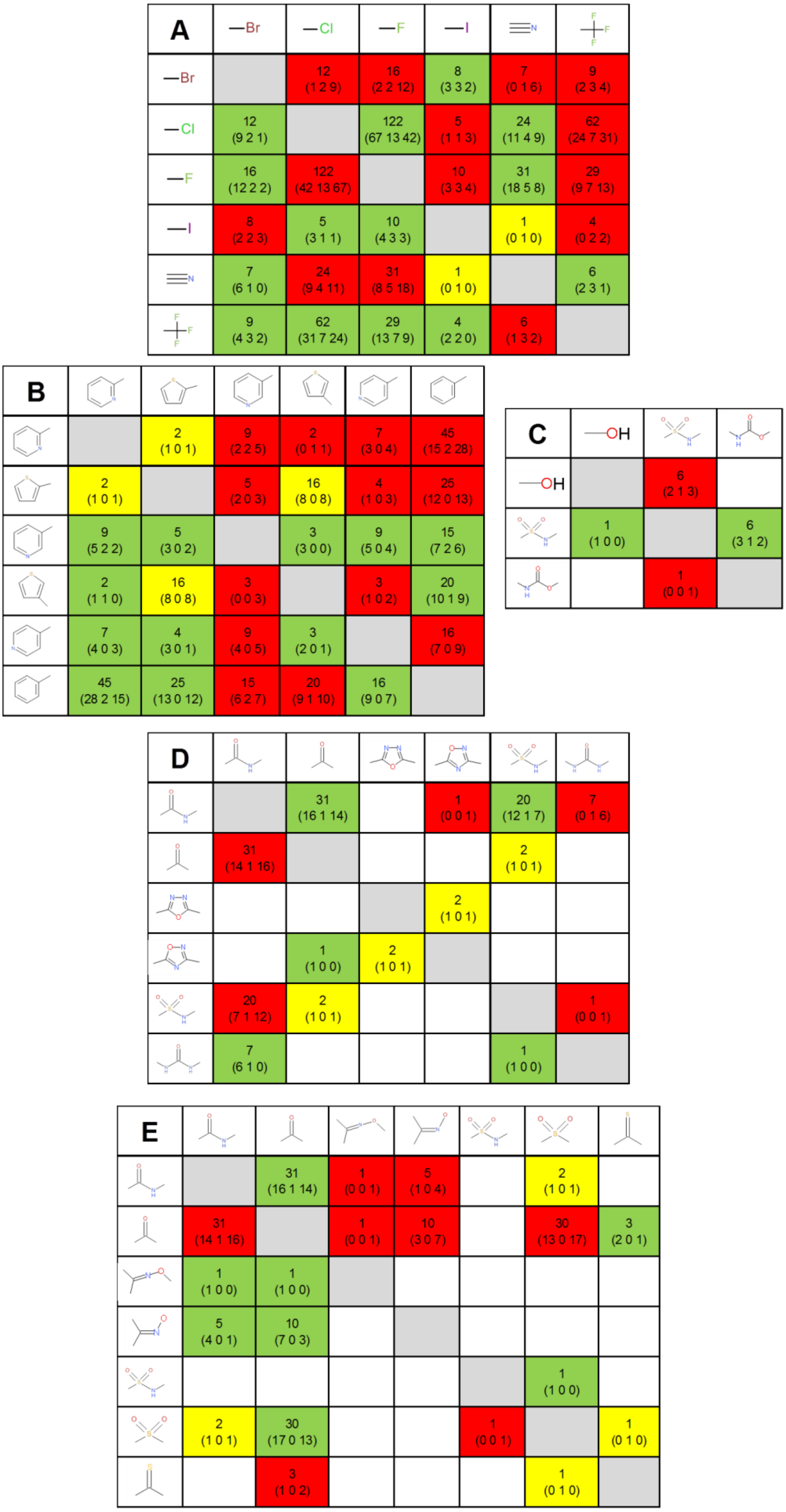

A	—Br	—Cl	—F	—I	≡N	CF3
—Br		12 (1 2 9)	16 (2 2 12)	8 (3 3 2)	7 (0 1 6)	9 (2 3 4)
—Cl	12 (9 2 1)		122 (67 13 42)	5 (1 1 3)	24 (11 4 9)	62 (24 7 31)
—F	16 (12 2 2)	122 (42 13 67)		10 (3 3 4)	31 (18 5 8)	29 (9 7 13)
—I	8 (2 2 3)	5 (3 1 1)	10 (4 3 3)		1 (0 1 0)	4 (0 2 2)
≡N	7 (6 1 0)	24 (9 4 11)	31 (8 5 18)	1 (0 1 0)		6 (2 3 1)
CF3	9 (4 3 2)	62 (31 7 24)	29 (13 7 9)	4 (2 2 0)	6 (1 3 2)	

B						
		2 (1 0 1)	9 (2 2 5)	2 (0 1 1)	7 (3 0 4)	45 (15 2 28)
	2 (1 0 1)		5 (2 0 3)	16 (8 0 8)	4 (1 0 3)	25 (12 0 13)
	9 (5 2 2)	5 (3 0 2)		3 (3 0 0)	9 (5 0 4)	15 (7 2 6)
	2 (1 1 0)	16 (8 0 8)	3 (0 0 3)		3 (1 0 2)	20 (10 1 9)
	7 (4 0 3)	4 (3 0 1)	9 (4 0 5)	3 (2 0 1)		16 (7 0 9)
	45 (28 2 15)	25 (13 0 12)	15 (6 2 7)	20 (9 1 10)	16 (9 0 7)	

C	—OH		
—OH		6 (2 1 3)	
	1 (1 0 0)		6 (3 1 2)
		1 (0 0 1)	

D						
		31 (16 1 14)		1 (0 0 1)	20 (12 1 7)	7 (0 1 6)
	31 (14 1 16)				2 (1 0 1)	
				2 (1 0 1)		
		1 (1 0 0)	2 (1 0 1)			
	20 (7 1 12)	2 (1 0 1)				1 (0 0 1)
	7 (6 1 0)				1 (1 0 0)	

E							
		31 (16 1 14)	1 (0 0 1)	5 (1 0 4)		2 (1 0 1)	
	31 (14 1 16)		1 (0 0 1)	10 (3 0 7)		30 (13 0 17)	3 (2 0 1)
	1 (1 0 0)	1 (1 0 0)					
	5 (4 0 1)	10 (7 0 3)					
						1 (1 0 0)	
	2 (1 0 1)	30 (17 0 13)			1 (0 0 1)		1 (0 1 0)
		3 (1 0 2)				1 (0 1 0)	

Figure S1. All bioisosteric replacements for 5-HT$_{1A}$R ligands belonging to: halogen (panel A), phenyl (B), hydroxyl (C), amide (D) and carbonyl (E) modifications. Total number of such replacements are given in the intersection field, along with the number of replacements which increase (X _ _), do not change (_ X _) and decrease (_ _ X). Desirable substitutions are backgrounded in green, ones decreasing the activity in red and not statistically influencing the activity in yellow.

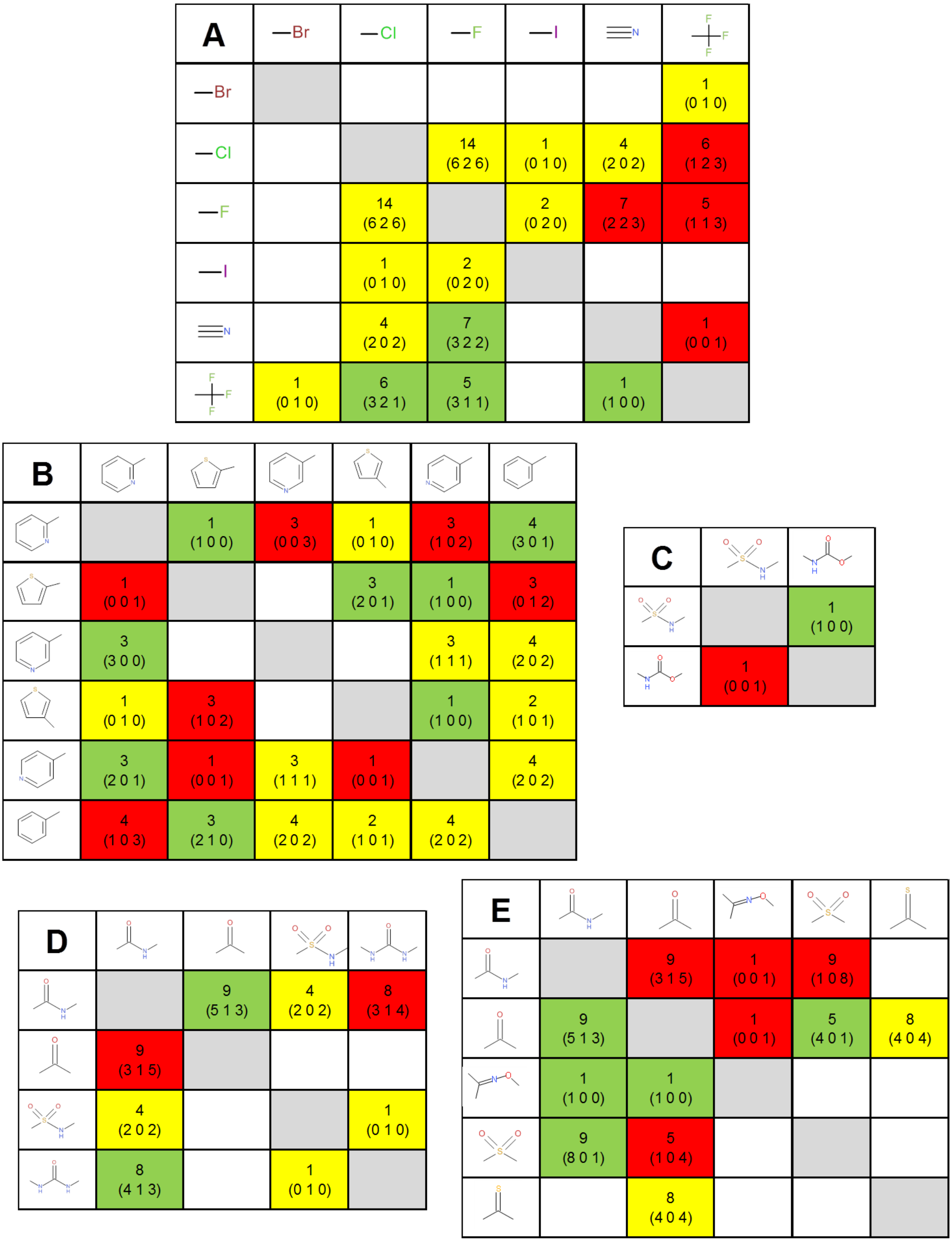

Figure S2. All bioisosteric replacements for 5-HT_{1B}R ligands belonging to: halogen (panel A), phenyl (B), hydroxyl (C), amide (D) and carbonyl (E) modifications. Total number of such replacements are given in the intersection field, along with the number of replacements which increase (X _ _), do not change (_ X _) and decrease (_ _ X). Desirable substitutions are backgrounded in green, ones decreasing the activity in red and not statistically influencing the activity in yellow.

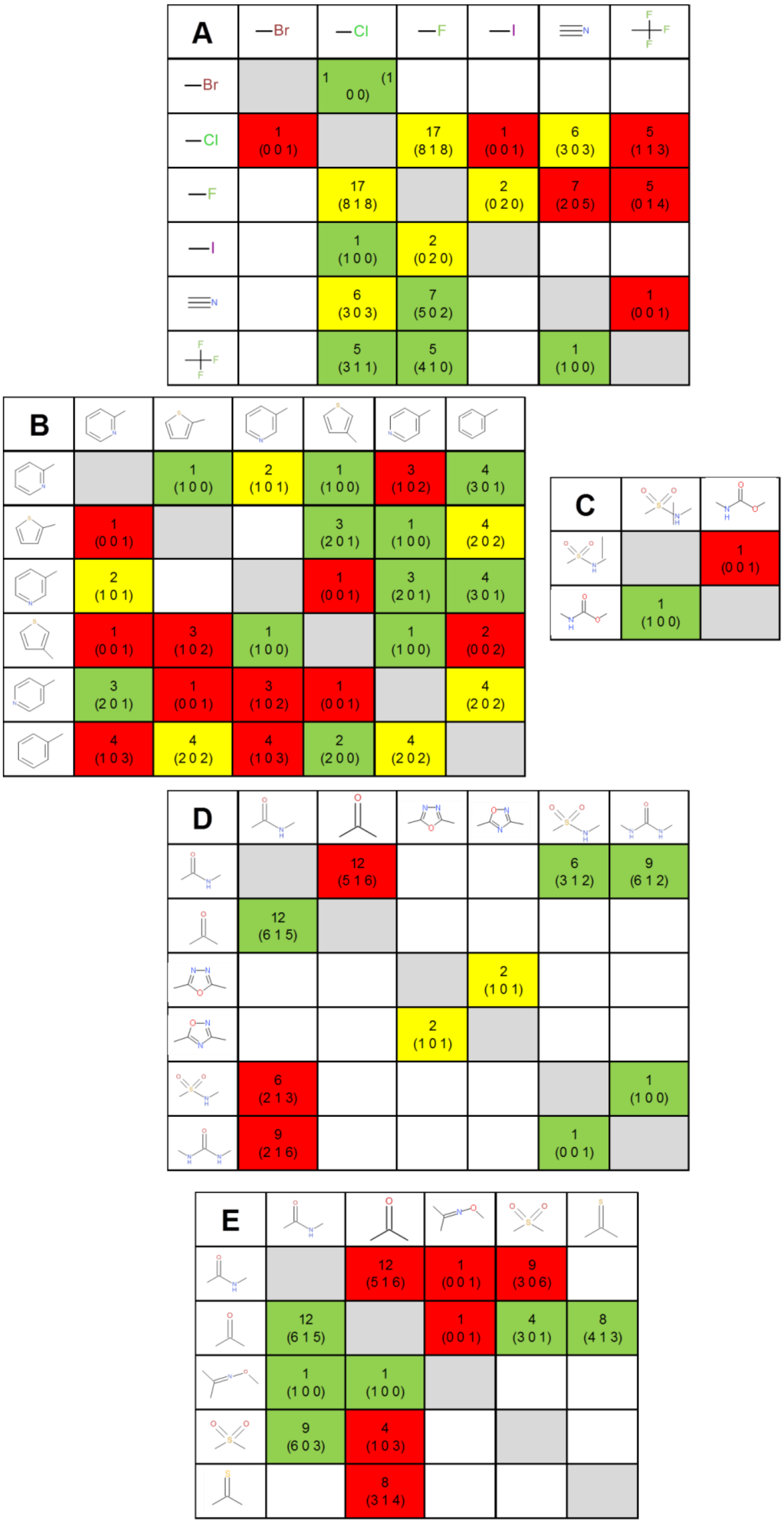

Figure S3. All bioisosteric replacements for 5-HT$_{1D}$R ligands belonging to: halogen (panel A), phenyl (B), hydroxyl (C), amide (D) and carbonyl (E) modifications. Total number of such replacements are given in the intersection field, along with the number of replacements which increase (X _ _), do not change (_ X _) and decrease (_ _ X). Desirable substitutions are backgrounded in green, ones decreasing the activity in red and not statistically influencing the activity in yellow.

A	—Cl	—F	—I	$-CF_3$
—Cl		3 (0 3 0)	1 (0 1 0)	1 (0 1 0)
—F	3 (0 3 0)		2 (0 2 0)	1 (0 1 0)
—I	1 (0 1 0)	2 (0 2 0)		
$-CF_3$	1 (0 1 0)	1 (0 1 0)		

Figure S4. All bioisosteric replacements for 5-HT$_{1E}$R ligands belonging to: halogen modifications. Total number of such replacements are given in the intersection field, along with the number of replacements which increase (X _ _), do not change (_ X _) and decrease (_ _ X). Desirable substitutions are backgrounded in green, ones decreasing the activity in red and not statistically influencing the activity in yellow.

A	—Cl	—F	≡N
—Cl		1 (0 0 1)	
—F	1 (1 0 0)		2 (1 1 0)
≡N		2 (0 1 1)	

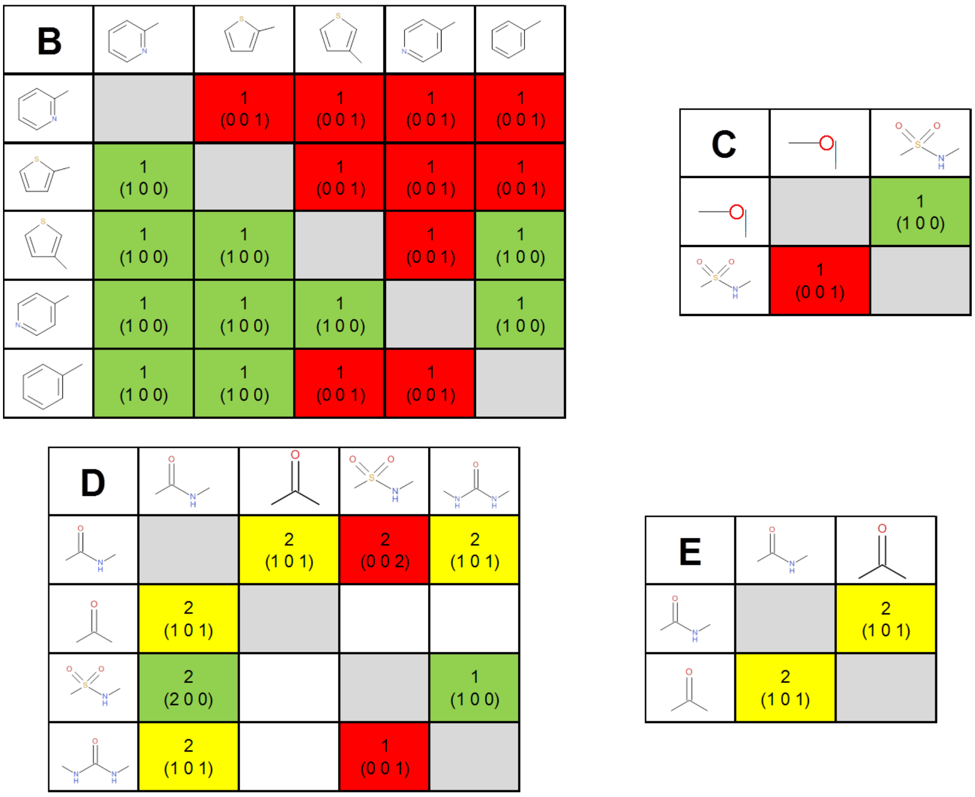

Figure S5. All bioisosteric replacements for 5-HT$_{1F}$R ligands belonging to: halogen (panel A), phenyl (B), hydroxyl (C), amide (D) and carbonyl (E) modifications. Total number of such replacements are given in the intersection field, along with the number of replacements which increase (X _ _), do not change (_ X _) and decrease (_ _ X). Desirable substitutions are backgrounded in green, ones decreasing the activity in red and not statistically influencing the activity in yellow.

.

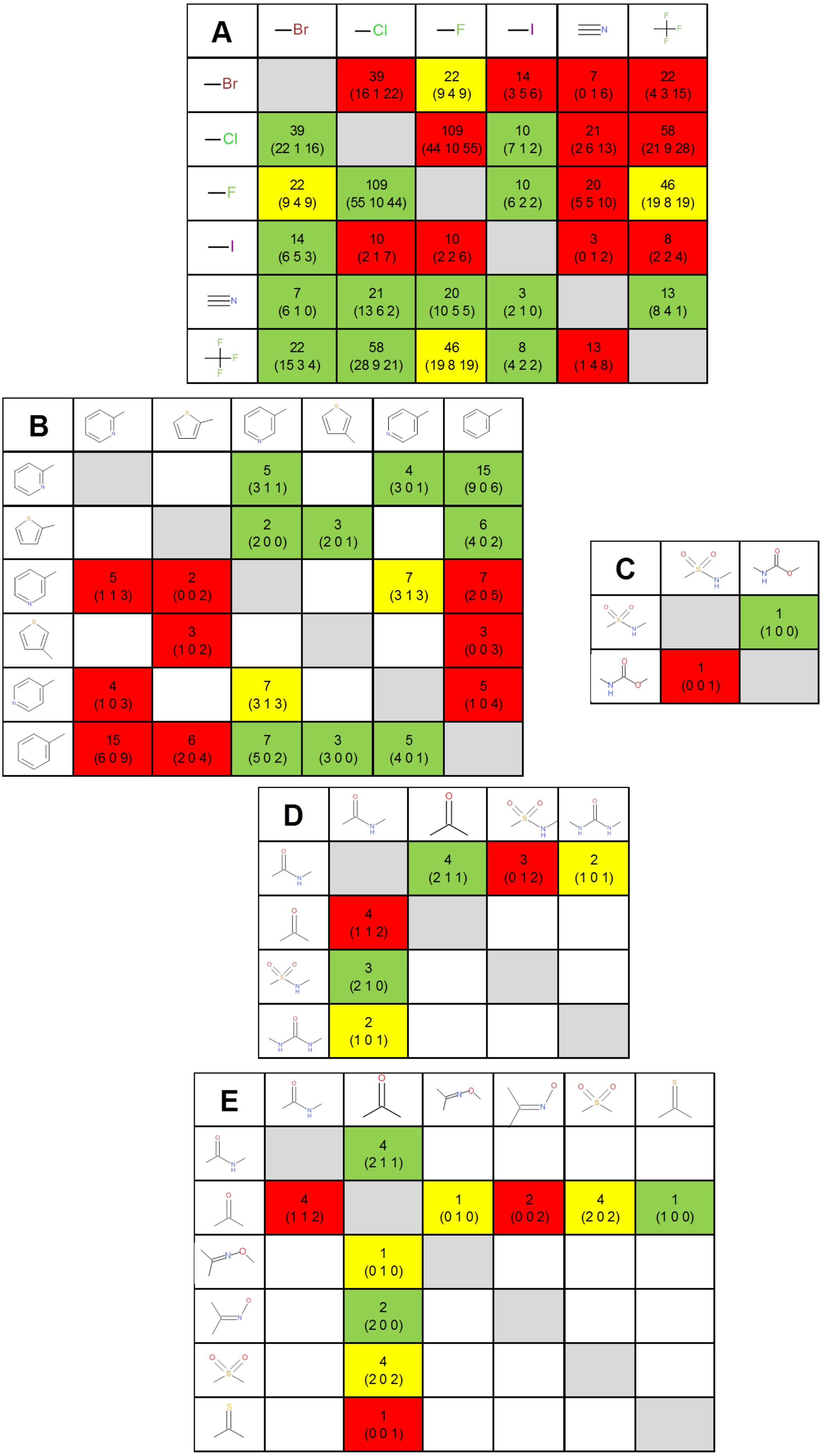

Figure S6. All bioisosteric replacements for 5-HT_{2A}R ligands belonging to: halogen (panel A), phenyl (B), hydroxyl (C), amide (D) and carbonyl (E) modifications. Total number of such replacements are given in the intersection field, along with the number of replacements which increase (X _ _), do not change (_ X _) and decrease (_ _ X). Desirable substitutions are backgrounded in green, ones decreasing the activity in red and not statistically influencing the activity in yellow.

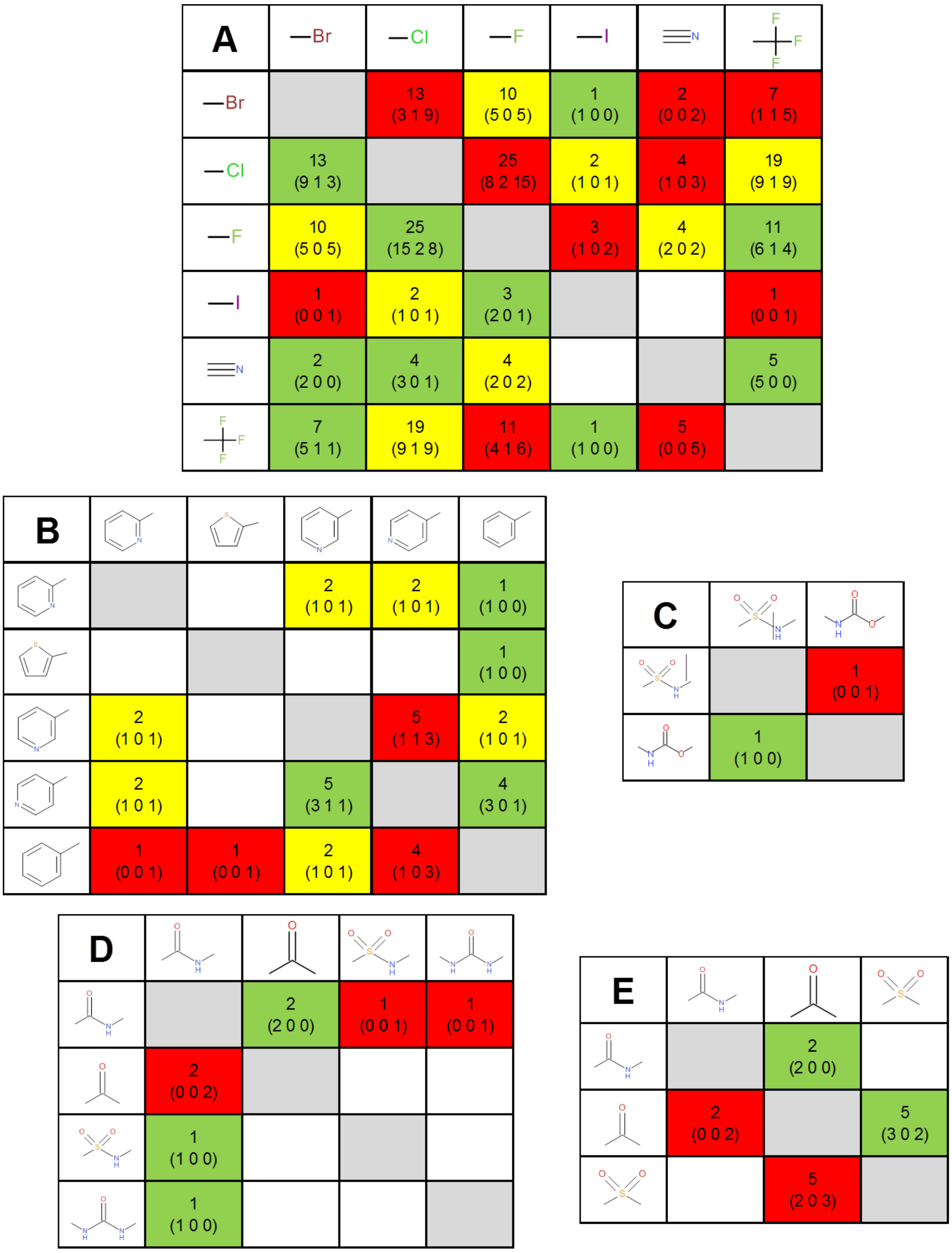

Figure S7. All bioisosteric replacements for 5-HT_{2B}R ligands belonging to: halogen (panel A), phenyl (B), hydroxyl (C), amide (D) and carbonyl (E) modifications. Total number of such replacements are given in the intersection field, along with the number of replacements which increase (X _ _), do not change (_ X _) and decrease (_ _ X). Desirable substitutions are backgrounded in green, ones decreasing the activity in red and not statistically influencing the activity in yellow.

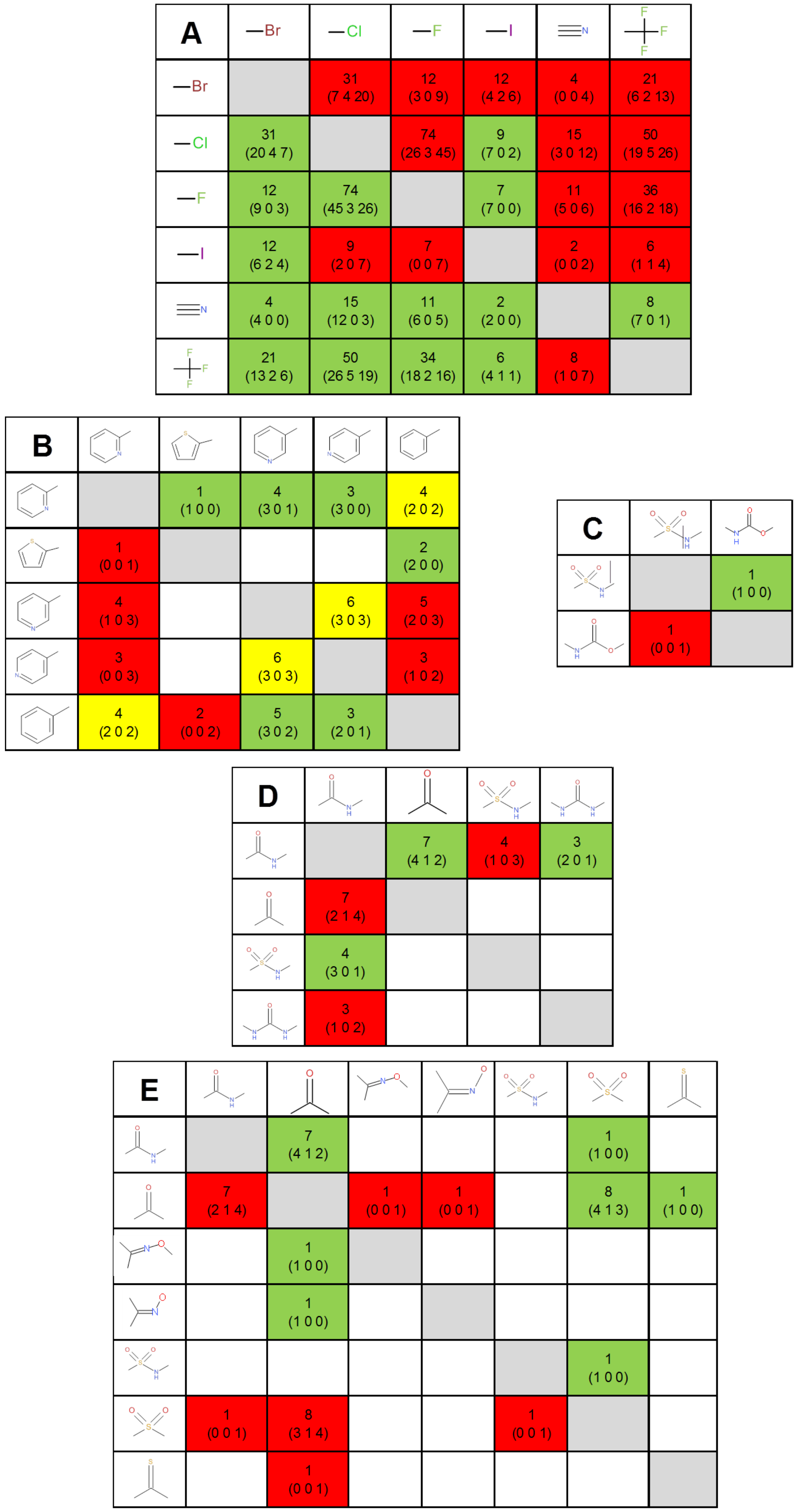

A	—Br	—Cl	—F	—I	≡N	—CF3
—Br		31 (7 4 20)	12 (3 0 9)	12 (4 2 6)	4 (0 0 4)	21 (6 2 13)
—Cl	31 (20 4 7)		74 (26 3 45)	9 (7 0 2)	15 (3 0 12)	50 (19 5 26)
—F	12 (9 0 3)	74 (45 3 26)		7 (7 0 0)	11 (5 0 6)	36 (16 2 18)
—I	12 (6 2 4)	9 (2 0 7)	7 (0 0 7)		2 (0 0 2)	6 (1 1 4)
≡N	4 (4 0 0)	15 (12 0 3)	11 (6 0 5)	2 (2 0 0)		8 (7 0 1)
—CF3	21 (13 2 6)	50 (26 5 19)	34 (18 2 16)	6 (4 1 1)	8 (1 0 7)	

B					
		1 (1 0 0)	4 (3 0 1)	3 (3 0 0)	4 (2 0 2)
	1 (0 0 1)				2 (2 0 0)
	4 (1 0 3)			6 (3 0 3)	5 (2 0 3)
	3 (0 0 3)		6 (3 0 3)		3 (1 0 2)
	4 (2 0 2)	2 (0 0 2)	5 (3 0 2)	3 (2 0 1)	

C		
		1 (1 0 0)
	1 (0 0 1)	

D				
		7 (4 1 2)	4 (1 0 3)	3 (2 0 1)
	7 (2 1 4)			
	4 (3 0 1)			
	3 (1 0 2)			

E							
		7 (4 1 2)				1 (1 0 0)	
	7 (2 1 4)		1 (0 0 1)	1 (0 0 1)		8 (4 1 3)	1 (1 0 0)
		1 (1 0 0)					
		1 (1 0 0)					
						1 (1 0 0)	
	1 (0 0 1)	8 (3 1 4)			1 (0 0 1)		
		1 (0 0 1)					

Figure S8. All bioisosteric replacements for 5-HT$_{2C}$R ligands belonging to: halogen (panel A), phenyl (B), hydroxyl (C), amide (D) and carbonyl (E) modifications. Total number of such replacements are given in the intersection field, along with the number of replacements which increase (X _ _), do not change (_ X _) and decrease (_ _ X). Desirable substitutions are backgrounded in green, ones decreasing the activity in red and not statistically influencing the activity in yellow.

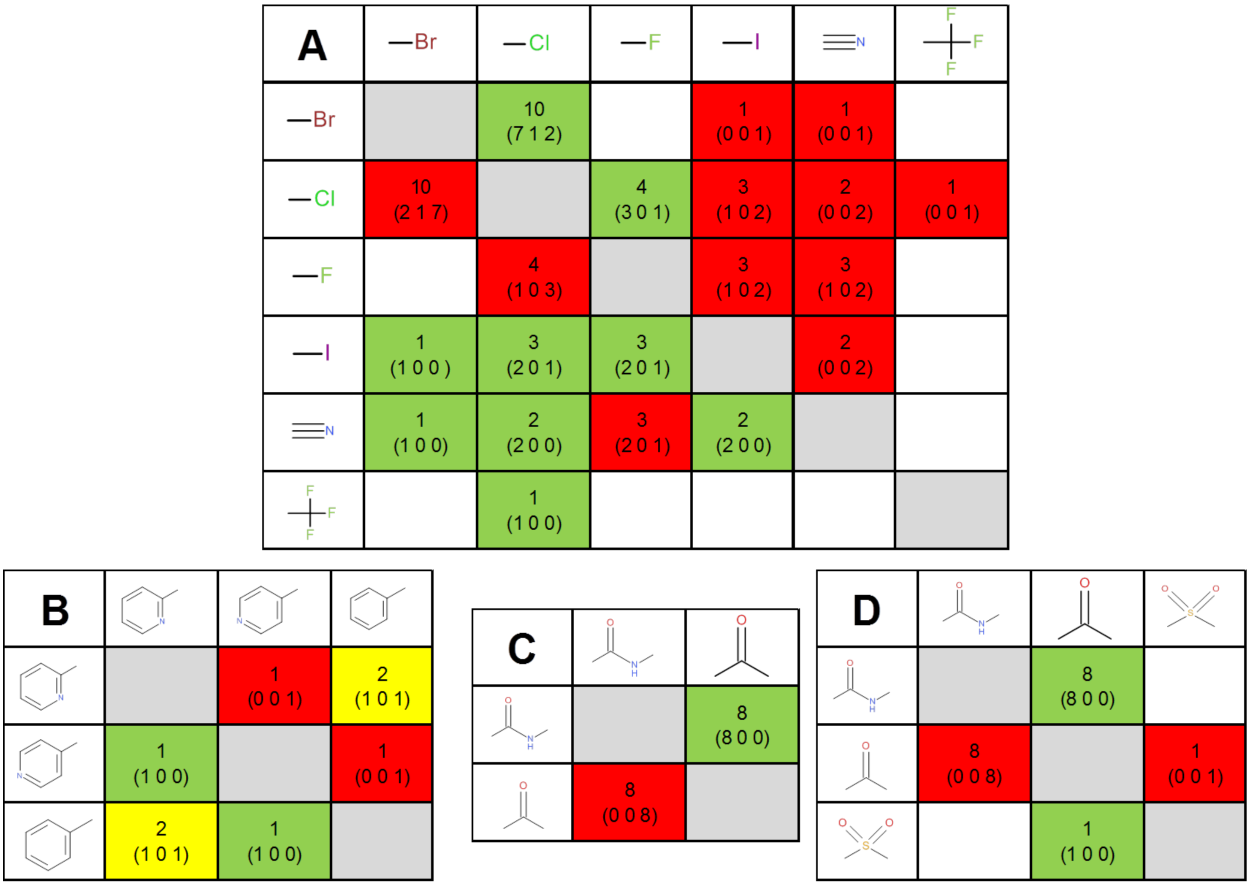

Figure S9. All bioisosteric replacements for 5-HT$_4$R ligands belonging to: halogen (panel A), phenyl (B), amide (C) and carbonyl (D) modifications. Total number of such replacements are given in the intersection field, along with the number of replacements which increase (X _ _), do not change (_ X _) and decrease (_ _ X). Desirable substitutions are backgrounded in green, ones decreasing the activity in red and not statistically influencing the activity in yellow.

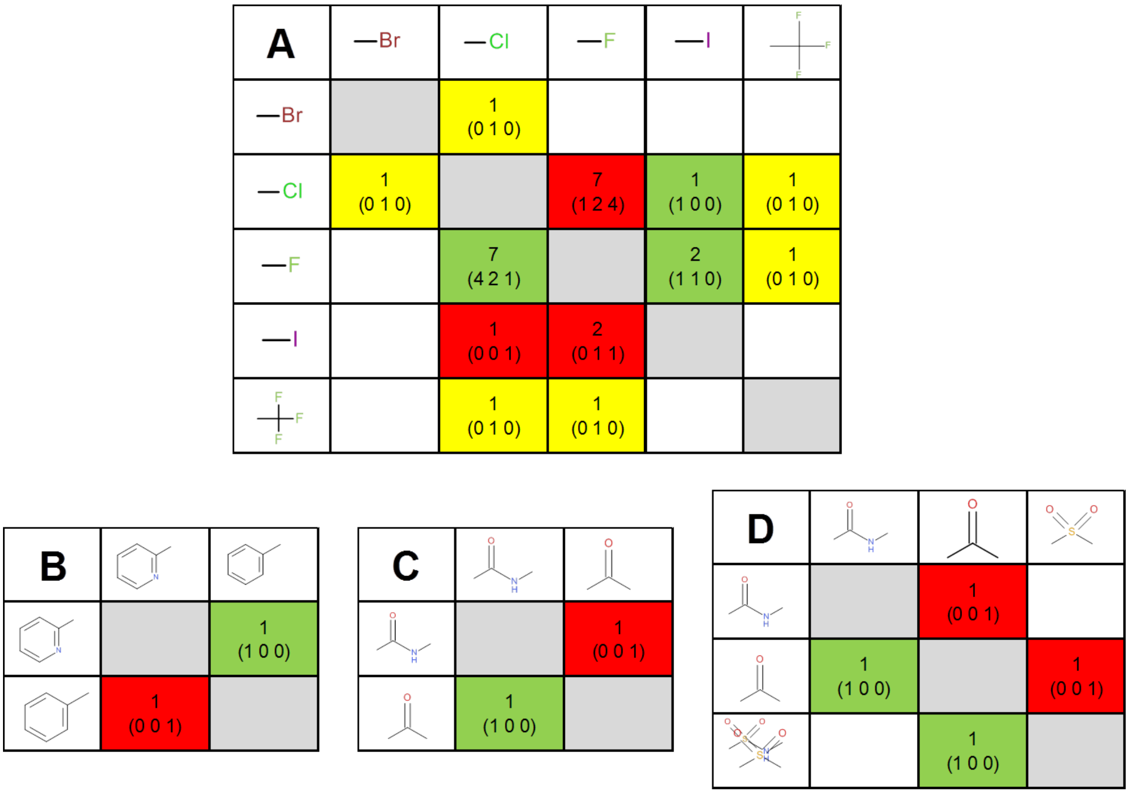

Figure S10. All bioisosteric replacements for 5-HT_{5A}R ligands belonging to: halogen (panel A), phenyl (B), amide (C) and carbonyl (D) modifications. Total number of such replacements are given in the intersection field, along with the number of replacements which increase (X _ _), do not change (_ X _) and decrease (_ _ X). Desirable substitutions are backgrounded in green, ones decreasing the activity in red and not statistically influencing the activity in yellow.

A	—Br	—Cl	—F	—I	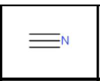	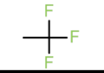
—Br		4 (1 1 2)	1 (0 0 1)			2 (1 0 1)
—Cl	4 (2 1 1)		35 (13 5 17)	1 (1 0 0)	3 (1 0 2)	12 (4 1 7)
—F	1 (1 0 0)	35 (17 5 13)		3 (3 0 0)	3 (1 0 2)	10 (3 1 6)
—I		1 (0 0 1)	3 (0 0 3)			
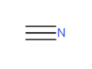		3 (2 0 1)	3 (2 0 1)			3 (1 0 2)
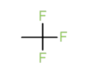	2 (1 0 1)	12 (7 1 4)	10 (6 1 3)		3 (2 0 1)	

B	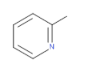	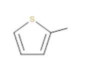	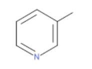	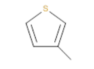	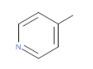	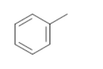
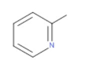		1 (1 0 0)	1 (0 0 1)		1 (0 0 1)	8 (6 0 2)
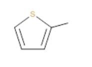	1 (0 0 1)		2 (0 0 2)	1 (0 0 1)	1 (0 0 1)	2 (1 0 1)
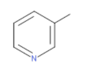	1 (1 0 0)	2 (2 0 0)		1 (1 0 0)	1 (1 0 0)	2 (2 0 0)
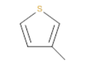		1 (1 0 0)	1 (0 0 1)			1 (1 0 0)
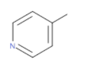	1 (1 0 0)	1 (1 0 0)	1 (0 0 1)			6 (5 0 1)
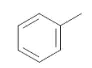	8 (2 0 6)	2 (1 0 1)	2 (0 0 2)	1 (0 0 1)	6 (1 0 5)	

C	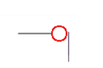	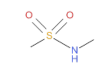	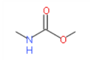
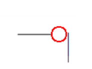		1 (0 0 1)	
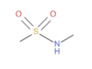	1 (1 0 0)		1 (1 0 0)
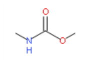		1 (0 0 1)	

D	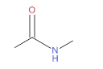	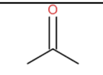	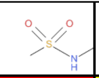	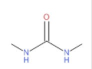
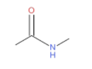		2 (1 1 0)	2 (0 0 2)	1 (0 1 0)
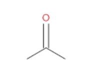	2 (0 1 1)		1 (0 0 1)	
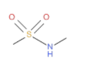	2 (2 0 0)	1 (1 0 0)		
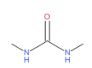	1 (0 1 0)			

E	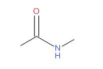	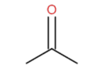	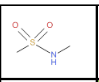	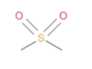
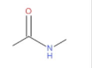		2 (1 1 0)		1 (1 0 0)
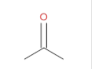	2 (0 1 1)			10 (1 3 6)
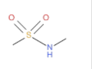				1 (1 0 0)
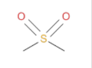	1 (0 0 1)	10 (6 3 1)	1 (0 0 1)	

Figure S11. All bioisosteric replacements for 5-HT$_7$R ligands belonging to: halogen (panel A), phenyl (B), hydroxyl (C), amide (D) and carbonyl (E) modifications. Total number of such replacements are given in the intersection field, along with the number of replacements which increase (X _ _), do not change (_ X _) and decrease (_ _ X). Desirable substitutions are backgrounded in green, ones decreasing the activity in red and not statistically influencing the activity in yellow.

Table S1. The most valuable substitutions for modifying ligands of one target to obtain promising ligands of another target. Only substitutions which appear at least five times in global analysis and with at least two time more examples of increasing affinity than decreasing (or vice versa) were taken into account.

Original target	Destination target	Transformation	No. of transformations	Increasing activity	No changes	Decreasing activity
5-HT_{1A}	5-HT_{1B}	Amide to Carbonyl	8	5	1	2
		Amide to Sulfonamide	5	5	0	0
		Carbonyl to Sulfone	5	1	0	4
		Chlorine to Fluorine	13	2	0	11
		Chlorine to Nitrile	6	2	0	4
		Contract Ring by One	18	5	3	10
		Expand Ring by One	16	3	0	13
		LinearABC to Ring5ABC	10	3	0	7
		Sulfone to Carbony	6	0	0	6
	5-HT_{1D}	Amide to Carbonyl	9	8	0	1
		Amide to Urea	9	8	1	0
		Chlorine to Nitrile	5	0	1	4
		Contract Ring by One	28	20	0	8
		Contract Ring by Two	6	4	0	2
		Sulfonamide to Amide	5	5	0	0
	5-HT_{1F}	Contract Ring by One	6	5	0	1
	5-HT_{2A}	2Pyridine to Phenyl	13	2	0	11
		Amide to Sulfonamide	5	0	0	5
		Bromine to Trifluoromethane	5	3	2	0
		Expand Ring by One	38	11	0	27
		LinearABC to Ring5ABC	9	2	0	7
		Phenyl to 3 Pyridine	5	4	0	1
		Trifluoromethane to Fluorine	17	3	7	7
	5-HT_{2B}	Contract Ring by One	9	2	0	7
		Expand Ring by One	9	2	0	7
	5-HT_{2C}	Chlorine to Fluorine	10	3	0	7
		Contract Ring by One	16	5	1	10
		Expand Ring by One	18	2	0	16
		Fluorine to Trifluoromethane	5	1	0	4
		LinearABC to Ring5ABC	6	2	0	4
		Phenyl to 2 Pyridine	5	1	0	4
		Trifluoromethane to Chlorine	8	5	1	2
		Trifluoromethane to Fluorine	5	1	1	3
	5-HT_6	Chlorine to Fluorine	10	3	0	7
		Contract Ring by One	11	3	0	8
		LinearABC to Ring5ABC	13	9	0	4
	5-HT_7	Amide to Carbonyl	7	1	0	6
		Contract Ring by One	24	7	1	16
		Fluorine to Chlorine	12	4	0	8
5-HT_{1B}	5-HT_{1D}	Amide to Carbonyl	10	9	0	1
		Amide to Sulfone	9	9	0	0

		Amide to Urea	9	9	0	0
		Carbonyl to Thiocarb	8	8	0	0
		Chlorine to Fluorine	14	10	2	2
		Contract Ring by One	19	16	0	3
		Expand Ring by One	19	16	0	3
		Fluorine to Chlorine	16	11	2	3
		Fluorine to Trifluoromethane	5	3	1	1
		LinearABC to Ring5ABC	14	11	0	3
		Nitrile to Fluorine	7	5	0	2
		Phenyl to 3 Pyridine	5	4	1	0
		Sulfone to Amide	9	8	0	1
		Thiocarb to Carbonyl	8	8	0	0
		Trifluoromethane to Chlorine	5	4	1	0
		Trifluoromethane to Fluorine	5	3	1	1
	5-HT_{1F}	Contract Ring by One	6	4	1	1
	5-HT_{2A}	Chlorine to Fluorine	7	1	2	4
		Contract Ring by One	7	5	0	2
	5-HT_{2B}	Contract Ring by One	8	6	0	2
	5-HT_{2C}	Chlorine to Fluorine	7	2	1	4
		Contract Ring by One	7	5	0	2
		Fluorine to Chlorine	8	2	1	5
	5-HT_6	Chlorine to Fluorine	8	2	2	4
		LinearABC to Ring5ABC	5	4	0	1
	5-HT_7	Chlorine to Fluorine	8	6	0	2
		Contract Ring by One	8	6	0	2
		Fluorine to Chlorine	7	5	0	2
5-HT_{1D}	5-HT_{2A}	Chlorine to Fluorine	9	2	1	6
		Expand Ring by One	5	0	0	5
	5-HT_{2B}	Expand Ring by One	6	2	0	4
	5-HT_{2C}	Contract Ring by One	5	0	0	5
		Expand Ring by One	7	2	0	5
	5-HT_6	Chlorine to Fluorine	10	2	2	6
		LinearABC to Ring5ABC	7	6	0	1
	5-HT_7	Chlorine to Fluorine	8	5	1	2
		Expand Ring by One	6	4	0	2
5-HT_{1E}	5-HT_{2A}	Expand Ring by One	5	4	0	1
	5-HT_7	Contract Ring by One	6	5	1	0
5-HT_{1F}	5-HT_6	LinearABC to Ring5ABC	5	4	0	1
	5-HT_7	Contract Ring by One	6	5	1	0
5-HT_{2A}	5-HT_{2B}	Bromine to Fluorine	6	1	1	4
		Chlorine to Bromine	8	7	1	0
		Chlorine to Trifluorimethane	20	14	2	4
		Expand Ring by One	25	6	1	18
		Fluorine to Bromine	6	5	0	1
		Fluorine to Chlorine	23	17	2	4
		LinearABC to Ring6ABC	6	6	0	0
		Nitrile to Trifluoromethane	6	6	0	0
		Trifluoromethane to Bromine	7	7	0	0

		Trifluoromethane to Chlorine	15	11	1	3
		Trifluoromethane to Fluorine	9	6	0	3
	5-HT$_{2C}$	3Pyridine to 4Pyridine	6	5	0	1
		4Pyridine to 3Pirydine	6	4	0	2
		Expand Ring by One	67	17	2	48
		Expand Ring by Two	6	2	0	4
		Fluorine to Bromine	12	8	0	4
		Fluorine to Nitrile	11	3	0	8
		Iodine to Fluorine	8	2	0	6
		LinearABC to Ring5ABC	9	1	0	8
		LinearABC to Ring6ABC	20	3	4	13
		Nitrile to Fluorine	13	4	0	9
		Nitrile to Trifluoromethane	8	7	0	1
		Sulfone to Carbonyl	7	1	0	6
		Trifluoromethane to Chlorine	38	27	1	10
		Trifluoromethane to Iodine	6	4	0	2
	5-HT$_6$	LinearABC to Ring5ABC	16	14	0	2
	5-HT$_7$	Chlorine to Fluorine	12	8	0	4
		Fluorine to Chlorine	11	8	0	3
		Fluorine to Trifluoromethane	6	1	0	5
5-HT$_{2B}$	5-HT$_{2C}$	Bromine to Chlorine	9	8	1	0
		Bromine to Fluorine	6	5	0	1
		Bromine to Trifluoromethane	12	8	0	4
		Chlorine to Bromine	9	8	1	0
		Chlorine to Fluorine	22	16	0	6
		Chlorine to Trifluorimethane	19	16	0	3
		Fluorine to Bromine	6	6	0	0
		Fluorine to Chlorine	22	19	0	3
		Fluorine to Trifluoromethane	12	11	0	1
		LinearABC to Ring6ABC	6	1	1	4
		Nitrile to Trifluoromethane	5	5	0	0
		Trifluoromethane to Bromine	8	8	0	0
		Trifluoromethane to Chlorine	18	15	0	3
		Trifluoromethane to Fluorine	9	8	0	1
	5-HT$_6$	Contract Ring by One	11	9	0	2
		Expand Ring by One	10	9	0	1
	5-HT$_7$	Contract Ring by One	8	6	0	2
5-HT$_{2C}$	5-HT$_6$	Chlorine to Trifluorimethane	6	4	0	2
		Expand Ring by One	6	4	0	2
		LinearABC to Ring5ABC	14	10	0	4
	5-HT$_7$	Contract Ring by One	11	10	0	1
		Expand Ring by One	9	6	0	3
		Fluorine to Chlorine	8	5	1	2
5-HT$_{5A}$	5-HT$_6$	LinearABC to Ring5ABC	7	6	0	1
5-HT$_6$	5-HT$_7$	2Thiophene to Phenyl	5	1	0	4
		Trifluoromethane to	6	2	0	4
